# Deciphering Phosphate Deficiency-Mediated Temporal Effects on Different Root Traits in Rice Grown in a Modified Hydroponic System

**DOI:** 10.3389/fpls.2016.00550

**Published:** 2016-05-04

**Authors:** Manisha Negi, Raghavendrarao Sanagala, Vandna Rai, Ajay Jain

**Affiliations:** National Research Centre on Plant Biotechnology, Lal Bahadur Shastri BuildingNew Delhi, India

**Keywords:** *Oryza sativa*, phosphate deficiency, aerated hydroponic system, root system architecture, Pi content, Pi starvation-responsive genes

## Abstract

Phosphate (Pi), an essential macronutrient for growth and development of plant, is often limiting in soils. Plants have evolved an array of adaptive strategies including modulation of root system architecture (RSA) for optimal acquisition of Pi. In rice, a major staple food, RSA is complex and comprises embryonically developed primary and seminal roots and post-embryonically developed adventitious and lateral roots. Earlier studies have used variant hydroponic systems for documenting the effects of Pi deficiency largely on primary root growth. Here, we report the temporal effects of Pi deficiency in rice genotype MI48 on 15 ontogenetically distinct root traits by using easy-to-assemble and economically viable modified hydroponic system. Effects of Pi deprivation became evident after 4 days- and 7 days-treatments on two and eight different root traits, respectively. The effects of Pi deprivation for 7 days were also evident on different root traits of rice genotype Nagina 22 (N22). There were genotypic differences in the responses of primary root growth along with lateral roots on it and the number and length of seminal and adventitious roots. Notably though, there were attenuating effects of Pi deficiency on the lateral roots on seminal and adventitious roots and total root length in both these genotypes. The study thus revealed both differential and comparable effects of Pi deficiency on different root traits in these genotypes. Pi deficiency also triggered reduction in Pi content and induction of several Pi starvation-responsive (PSR) genes in roots of MI48. Together, the analyses validated the fidelity of this modified hydroponic system for documenting Pi deficiency-mediated effects not only on different traits of RSA but also on physiological and molecular responses.

## Introduction

Rice, a major staple food in Asia, is grown largely under rain-fed ecosystem on soils that are naturally low in phosphorus (P) (Gamuyao et al., 2012). P is an essential macronutrient required for growth and development of plant (López-Arredondo et al., 2014). Root system plays a key role in acquisition of inorganic phosphate (Pi); a readily bioavailable source of P in rhizosphere (Marschner, 1995). However, Pi is often limiting due to its slow diffusion rate and interactions with different soil constituents (Raghothama, 1999). Therefore, soil exploration by roots is critically important for optimal acquisition of Pi (Lynch, 2015).

*Arabidopsis thaliana*, a favored model plant species, has been extensively used for elucidation of Pi deficiency-mediated responses of root system architecture (RSA; Sánchez-Calderón et al., 2005; Gruber et al., 2013; Kellermeier et al., 2014). Conventionally, effects of Pi deprivation on RSA are documented by either growing on agar plate (Williamson et al., 2001; López-Bucio et al., 2002; Kellermeier et al., 2014) or hydroponically (Jain et al., 2009; Alatorre-Cobos et al., 2014) under aseptic condition. Pi deficiency induces inhibitory effects on the developmental responses of both embryonically and post-embryonically developed primary and lateral roots, respectively (Sánchez-Calderón et al., 2005; Jain et al., 2007).

Root system of *Oryza sativa* (rice) is relatively complex, comprising embryonically developed primary and seminal roots with post-embryonic adventitious roots making up bulk of the root system (Hochholdinger and Zimmermann, 2008). While primary and seminal roots play important roles during seedling stage, adventitious roots dominate the functional root system in mature plant (Hochholdinger et al., 2004). Different types of hydroponic system have been used for determining the effects of Pi deficiency on root development (Yi et al., 2005; Zhou et al., 2008; Dai et al., 2012, 2016; Wang et al., 2015). The effects of Pi deprivation have largely been focused on only a few root traits, i.e., total root length (Yi et al., 2005), primary root length (Shimizu et al., 2004; Zhou et al., 2008; Zheng et al., 2009; Hu et al., 2011; Dai et al., 2012, 2016; Wang S. et al., 2014; Wang et al., 2015), lateral root number on primary root (Wang S. et al., 2014), lateral root length (Yang et al., 2014), seminal root length (Ogawa et al., 2014); and number and/or length of adventitious roots (Zhou et al., 2008; Hu et al., 2011; Dai et al., 2012, 2016; Wang et al., 2015). None of these studies provided a holistic overview of the effects of Pi deficiency on different root traits. Different concentrations of Pi considered as P+ and P- media, variation in the duration of Pi deficiency treatment and use of different rice genotypes in these studies further makes it difficult to draw any explicit conclusion on the global effects of Pi deprivation on the developmental responses of ontogenetically distinct root traits.

In this study, we used modified hydroponic system for deciphering the effects of Pi deficiency on the developmental responses of primary, seminal and adventitious roots and also of lateral roots on each of them in rice *cv.* MI48 and N22. The modified hydroponic system was equally efficient for generating tissues for elucidation of Pi deficiency-mediated physiological and molecular responses.

## Materials and Methods

### Plant Material and Seed Germination

Seeds of rice (*O. sativa* L. ssp. *indica*) genotype MI48 and Nagina 22 (N22) were used for this study. In Petri plate (110 mm × 25 mm), lined with filter paper and wetted with sterile water, 10 seeds were placed equi-distant and wrapped in aluminum foil for maintaining dark condition. For each experiment, about 150–200 seeds were used. Petri plates were then placed in an incubator set at 28°C for 4 days. After germination, seedlings were transferred to Petri plate containing 1% (w/v) agar and scanned at 600 dots per inch (dpi) by using a desktop scanner. Scanned images were then used for documenting the radicle length by using ImageJ; a Java image-processing program (^1^Collins, 2007). Seedlings often show significant variation in their radicle length. Therefore, for minimizing the effects of intrinsic variability on subsequent treatment under different Pi regime, only those seedlings with radicle length in the range of 2–3 cm were selected.

### Modified Hydroponic System

Autoclavable hydroponic system was assembled by easily available components, i.e., a standard polycarbonate transparent Magenta (GA-7) box (width × length × height = 75 mm × 74 mm × 138 mm), support made of polycarbonate sheet (0.030″ thick), a polypropylene mesh (250 μm mesh size, width × length = 24″ × 12″ by Small Parts and available at amazon.com), aquarium air pump (power 5 W and pressure 2 MPa × 0.02 MPa), flexible air line tubing (3 mm in diameter) and tee connector. Polycarbonate sheets were cut into 80 mm × 40 mm rectangular pieces and notched at midpoint up to 20 mm so that the two pieces could fit together into an X-shaped support. Polypropylene mesh sheet was cut into a square piece (50 mm × 50 mm) and four holes (4 mm in diameter) were punched toward the perimeter for facilitating the penetration of radicle through the mesh into the nutrient medium. For experiments where rice seedlings are to be grown for a longer duration, the height of the X-shaped support could be easily increased up to 120 mm to ensure that root tip does not come in contact with the bottom of the Magenta box. Also, the number of seedlings in each magenta box could be reduced from four to a lesser number by punching the required number of holes in the mesh. Wedge support was placed into the Magenta box, filled with enough deionized water so that the level remained above the X-shaped wedge support and autoclaved. After autoclaving, water was removed from the Magenta box. To avoid warping, cut mesh pieces (10–15) were stacked and wrapped in aluminum foil and autoclaved separately. On each mesh, four germinated seedlings were placed close to the hole to facilitate penetration of radicle through the mesh and lowered gently on the wedge support placed in the Magenta box. Nutrient medium was then added (about 200 ml) to the hydroponic system to ensure that its level remained 2–3 mm above the X-shaped wedge support. Nutrient media (P+ and P-) were prepared as described (Jia et al., 2011) and buffered to pH 5.7 with 0.5 mM 2-(*N*-morpholino) ethanesulfonic acid (MES). P+ and P- represented 0.3 mM NaH_2_PO_4_ and 0 mM NaH_2_PO_4_, respectively. Hydroponic system was placed under controlled growth condition in the greenhouse (16-h day/8-h night cycle, 28 ± 2°C and relative humidity was maintained at ∼60–70%).

### Quantification of Total Shoot Area and RSA Parameters

**FIGURE 1:**
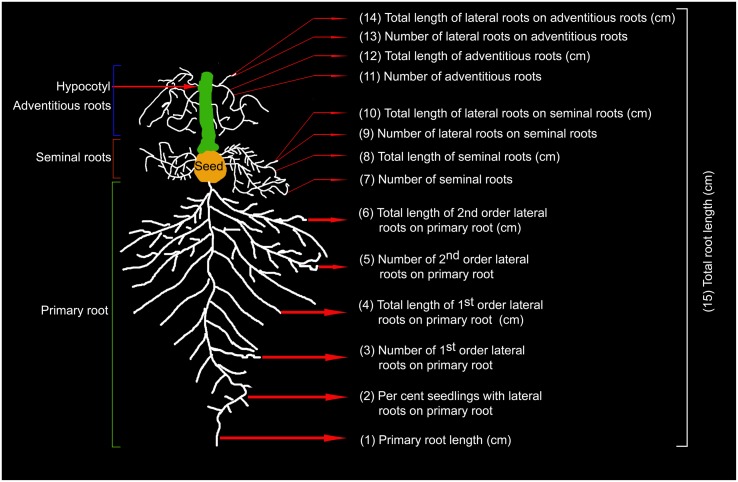
**Schematic overview of rice RSA.** Temporal effects of Pi deficiency was quantified on developmental responses of 15 roots traits comprising primary, seminal and adventitious roots and lateral roots on each of them.

Seedlings grown under P+ and P- conditions in the hydroponic system were removed along with the mesh sequentially 2, 4, and 7 days after treatment. Seedlings were transferred in an inverted position to a Petri plate containing pool of water. Under stereomicroscope, root and shoot were separated at shoot:hypocotyl junction. Carefully, primary, seminal and adventitious roots along with their lateral roots were separated from each other. Dissected roots were then transferred immediately to 70% (v/v) ethanol to avoid any desiccation and for subsequent documentation. Shoots were gently spread and pasted with glue stick on white sheet of paper. For revealing RSA, dissected roots were transferred from 70% (v/v) ethanol to a Petri plate containing 1% (w/v) agar. Lateral roots on primary, seminal and adventitious roots were spread gently with a camel hair brush under stereomicroscope ensuring no overlap. Glued shoots on paper and spread out roots on agar plates were scanned at 600 dpi using a desktop scanner. Scanned images were used for documenting 15 different RSA parameters (**Figure 1**) and total shoot area by using ImageJ program.

### Soluble Pi Content

Harvested roots were rinsed 5–6 times in deionized water, blot-dried gently, frozen in liquid nitrogen and ground to a fine powder and stored at -80°C till further use. Ground tissue (25–50 mg) was homogenized with 250 μl of 1% (v/v) glacial acetic acid, vortexed and centrifuged at 10,000 rpm for 5 min. Supernatant was collected for assaying Pi content by phosphomolybdate colorimetric assay as described (Ames, 1966). A standard curve generated with KH_2_PO_4_ was used for determining the concentration of soluble Pi.

### Real-Time PCR

The root samples collected from two independent biological experiments were pooled for isolating total RNA by using Spectrum^TM^ Plant Total RNA kit as described (Sigma, USA). DNase treatment was given for removing trace amount of DNA. RNA was quantified by NanoDrop 1000 Spectrophotometer (Thermo Scientific, USA) and its quality was assessed on 1.2% (w/v) denatured agarose gel. First-strand cDNA was synthesized from the total RNA (1 μg) using SuperScript^®^ III first-strand synthesis system (Invitrogen, USA). Real-time PCR was performed on Stratagene MX 3005P (Agilent Technologies, USA) using SYBR GreenER^TM^ qPCR Universal SuperMix (Invitrogen, USA). Gene-specific primers were designed using PrimerQuest software^2^ *OsRubQ1* was used as an internal control. Amplicons were subjected to meltcurve analysis for checking the specificity of amplified products. Relative expression levels of the genes were computed by 2^-ΔΔ^*^C^*_T_ method of relative quantification (Livak and Schmittgen, 2001). List of primers used for real-time PCR is given in Supplementary Table S1.

### Statistics

For each experiment, data were collected from 2 to 3 independent biological experiments. Statistical significance of differences between mean values was determined using Student’s *t*-test. Different letters on histograms were used for indicating means that were statistically different at *P* < 0.05.

## Results and Discussion

### Selection of Seedlings Prior to Treatment under Different Pi Regime

**FIGURE 2:**
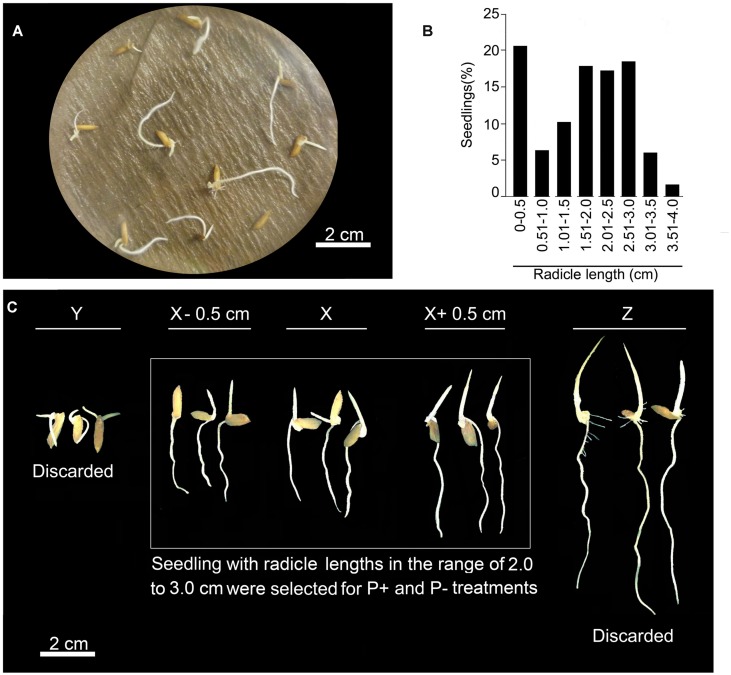
**Elimination of intrinsic variability in radicle length. (A)** Seeds of rice genotype MI48 were germinated in a Petri plate lined with wet filter paper at 28°C for 4 days in dark. **(B)** Radicle lengths of germinated seedlings were measured by ImageJ program and categorized into different size ranges of 0.5 cm each. Histogram represents per cent seedlings in different size ranges. **(C)** About 30–40% seedlings falling in the size range of 2.0–3.0 cm were selected and transferred to hydroponic set up for temporal treatment under P+ and P- conditions.

Radicle length of germinated rice seedlings varies significantly across different genotypes. For Pi deficiency treatment, uniformly grown seedlings are normally selected based on eyeballing, which could often lead to an erroneous selection. Therefore, to minimize the effects of intrinsic variability on radicle length during subsequent Pi deficiency treatment, a more pragmatic approach was adopted. Around 200 seeds were distributed uniformly in Petri plates (10 seeds/Petri plate) lined with wet filter paper and kept for germination at 28°C for 4 days (**Figure 2A**). Germinated seedling was transferred to 1% (w/v) agar plate and scanned. Scanned image was used for measuring radicle length using ImageJ program. Based on radicle length, seedlings were grouped into different size ranges of 0.5 cm each and computed per cent seedlings falling in each of these groups (**Figure 2B**). Radicles of several seedlings (∼20%) exhibited stunted growth with their lengths falling in the range of 0–0.5 cm. Per cent seedlings with radicle length in other size categories varied from ∼2 to 18%. It was interesting to note that ∼5% seedlings revealed an exaggerated radicle growth (∼3–4 cm). It was evident from this analysis that extensive variation in radicle length of rice seedlings could exert significant erroneous influence on Pi deficiency-mediated effects on different root traits. To circumvent this problem, only those seedlings were selected whose radicle length was in the range of 2–3 cm (**Figure 2C**). Rest of the seedlings outside this size range was discarded. Although, several studies have reported the effects of Pi deprivation on different root traits (Shimizu et al., 2004; Zhou et al., 2008; Dai et al., 2012; Ogawa et al., 2014; Yang et al., 2014), it is not evident from any of these studies as how the likely erroneous influence of intrinsic variability in radicle length of rice seedlings prior to P+ and P- treatments was addressed.

### Modified Hydroponic System

Conventionally rice is grown in a hydroponic system maintained under green house condition for deciphering Pi deficiency-mediated effects on the developmental responses of different root traits (Zhou et al., 2008; Dai et al., 2012; Yang et al., 2014). However, nutrient-rich medium of hydroponic system is often susceptible to elemental contamination, which often results in erroneous interpretations on the effect of Pi deficiency on various morphophysiological and molecular traits (Jain et al., 2009). In addition, growth of algal bloom, fungi, and bacteria in the medium aggravates the problem.

**FIGURE 3:**
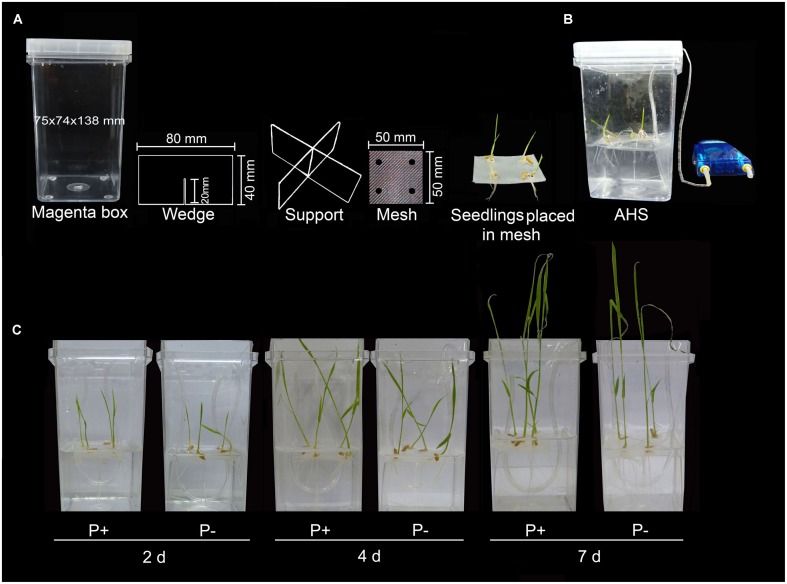
**Modified hydroponic system. (A)** Modified hydroponic system made of autoclavable Magenta box, polycarbonate wedge support, polypropylene mesh and germinated rice seedlings placed on the mesh with radicle traversing through the hole punched around its perimeter. **(B)** Complete aerated hydroponic system (AHS). **(C)** Seedlings were grown in AHS under P+ (0.3 mM NaH_2_PO_4_) and P- (0 mM NaH_2_PO_4_) conditions for 2, 4, and 7 days.

To circumvent these multitude of problems, hydroponic system was modified for growing rice under P+ and P- conditions by assembling easily available autoclavable components (Magenta box, polycarbonate X-shaped wedge support and polypropylene mesh; **Figure 3A**). Further, hydroponic set-up was aerated using aquarium air pump for proper oxygenation and nutrient circulation (**Figure 3B**). Non-aerated hydroponic system could limit oxygen availability to plant roots, which could trigger ethylene production and may exert adverse affects on root growth (Barrett-Lennard and Dracup, 1988). Modified hydroponic system was used for studying the temporal (2, 4, and 7 days) effects of Pi deficiency on morphophysiological and molecular responses (**Figure 3C**).

### Pi Deficiency-Mediated Affects on Phenotypic Traits

**FIGURE 4:**
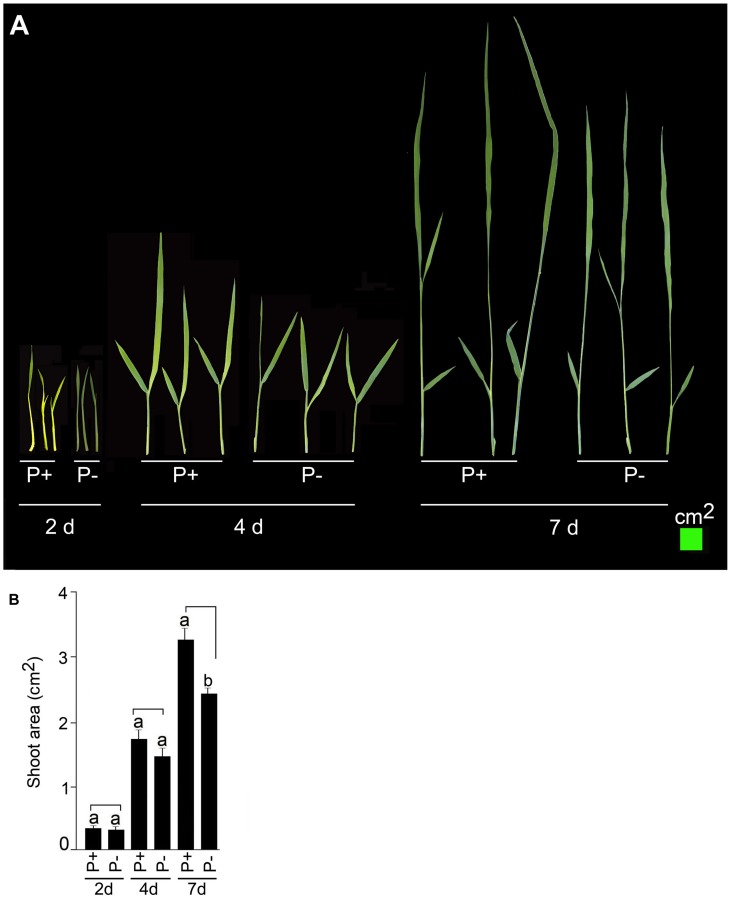
**Temporal effect of Pi deficiency on shoot area.** MI48 seedlings (4-days-old) were grown under P+ and P- conditions for 2, 4, and 7 days. **(A)** Harvested shoots were gently spread and scanned to reveal the details of leaves. **(B)** Data presented for total shoot area. Values are mean ± SE and different letters indicate that the means differ significantly (*P* < 0.05).

Temporal effects of Pi deficiency was determined on shoot phenotype and its total area (**Figure 4**). Pi deprivation for 2 and 4 days did not exert any significant (*P* < 0.05) influence on shoot phenotype (**Figure 4A**) and its total area (**Figure 4B**). The effect of Pi deficiency became evident on shoot growth only after 7 days treatment. Growth of P+ shoot was more vigorous compared with P- shoot (**Figure 4A**) and also area of P- shoot was ∼25% lower compared with P+ (**Figure 4B**). The result was consistent with an earlier study, which also showed attenuating effect of Pi deprivation on shoot length in rice (Yang et al., 2014). This suggested the suitability of modified hydroponic system for generating shoot tissues for Pi deficiency-mediated responses.

**FIGURE 5:**
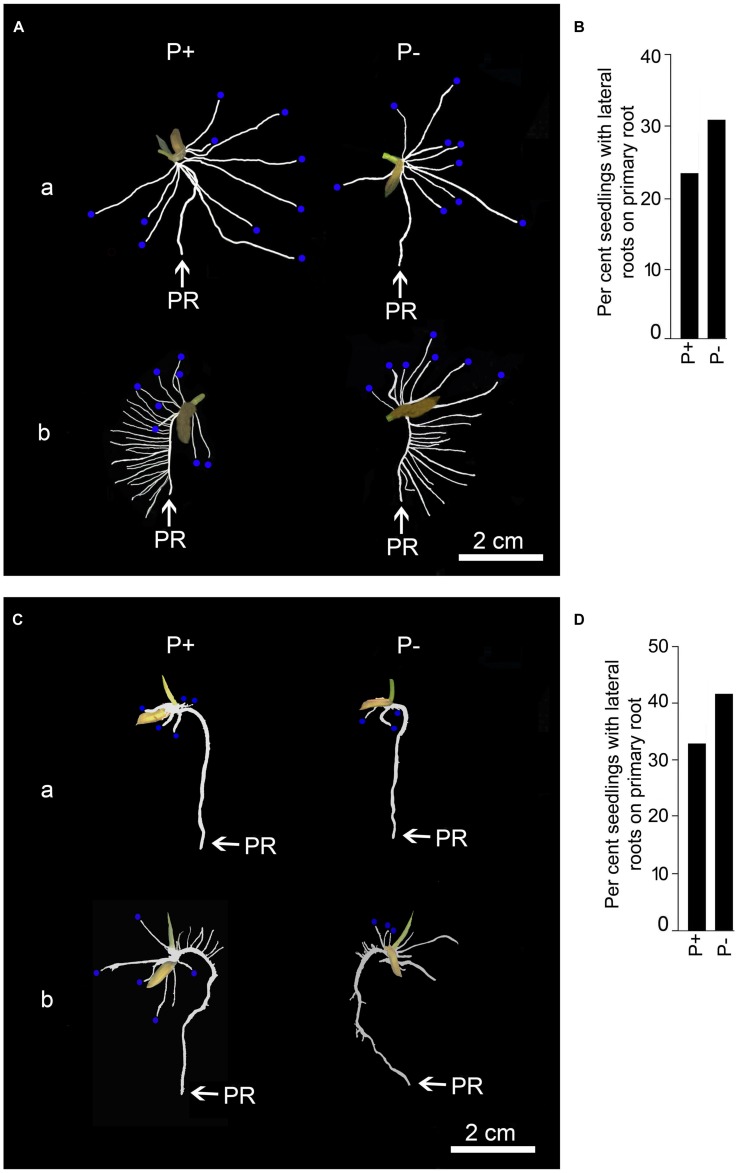
**Effects of Pi deficiency for 2 days on RSA under different growth conditions.** MI48 seedlings (4-days-old) were grown under P+ and P- conditions for 2 days in a modified hydroponic system as shown in **Figure 3C** and in Petri plates lined with blotting paper. **(A,C)** Harvested roots were spread to reveal the details of RSA showing (a) lack and (b) exuberant growth of lateral roots on primary root of P+ and P- seedlings during growth on modified hydroponic system **(A)** and in Petri plates lined with blotting paper **(C).** Seminal roots **(A,C)** are indicated with blue dots at their tips. **(B,D)** Data presented for per cent seedlings showing lateral root development on primary root during growth on modified hydroponic system **(B)** and in Petri plates lined with blotting paper **(D).**

Temporal effects of Pi deprivation was investigated on the responses of embryonically (primary and seminal) and post-embryonically (adventitious and lateral) developed roots. Two distinct root phenotypes were observed for both P+ and P- seedlings grown for 2 days (**Figure 5**). Although, majority of the primary roots of P+ and P- seedlings did not show lateral root growth (**Figure 5Aa**), 25–30% of them revealed their development (**Figure 5Ab**). However, there were significant variations in both the number (P+, 2.58 ± 1.71 [SE]; P-, 4.01 ± 2.09 [SE]) and total length (P+, 2.98 cm ± 1.85 cm [SE]; P-, 4.09 cm ± 2.07 cm [SE]) of these lateral roots. This highlighted the prevalence of extensive variability in the developmental responses of lateral roots on primary root irrespective of Pi regime. There was no significant (*P* < 0.05) difference in primary root length of P+ and P- seedlings and was comparable to the radicle length before the treatment (**Figure 2C**), which indicated no significant (*P* < 0.05) increment in this root trait during 2 days treatment. There was thus an apparent lack of correlation between growth responses of primary root and occasional post-embryonically developed lateral roots on them in P+ and P- seedlings. Number (P+, 7.01 ± 0.81 [SE]; P-, 6.75 ± 0.48 [SE]) and length (P+, 10.57 cm ± 1.85 cm [SE]; P-, 10.95 cm ± 1.14 cm [SE]) of seminal roots varied but the effect of Pi deficiency was not apparent. Neither lateral roots on seminal roots nor adventitious roots in P+ and P- seedlings could be detected after 2 days treatment.

To ensure that the responses of the root system under P+ and P- conditions in the modified hydroponic system was not an artifact, MI48 seedlings with radicle length in the range of 2–3 cm (**Figure 2C**) were also grown on square Petri plate (115 mm × 115 mm) lined with blotting paper kept moist with these nutrient solutions for 2 days. **Figure 5C** presents the RSA of these seedlings. Differences in primary root length of P+ (2.14 cm ± 0.06 cm [SE]) and P- (2.21 cm ± 0.06 cm [SE]) seedlings were insignificant (*P* < 0.05) and also the corresponding values were comparable with those grown in the modified hydroponic system. About 33% of both P+ and P- seedlings developed lateral roots (**Figure 5D**). Sporadic development of lateral roots on P+ and P- primary roots on blotting paper was similar to that observed in P+ and P- seedlings grown in the modified hydroponic system (**Figure 5B**). These lateral roots exhibited significant variations in both the number (P+, 3.08 ± 1.43 [SE]; P-, 4.75 ± 1.81 [SE]) and total length (P+, 0.48 cm ± 0.27 cm [SE]; P-, 0.56 cm ± 0.28 cm [SE]); a feature also observed with the seedlings grown in the modified hydroponic system. Overall, these root traits (primary root length, number and length of lateral roots) of MI48 showed comparable responses irrespective of Pi regime or growth conditions (hydroponics and blotting paper). This suggested that developmental responses of these root traits under P+ and P- conditions are not artifacts of the modified hydroponic system. Pi deficiency also did not exert any significant (*P* < 0.05) influence on the number (P+, 4.25 ± 0.65 [SE]; P-, 4.17 ± 0.49 [SE]) and total length (P+, 1.33 cm ± 0.29 cm [SE]; P-, 1.19 cm ± 0.19 cm [SE]) of seminal roots during growth on the blotting paper. Although, the developmental responses of seminal roots were not influenced by the Pi regime during growth in the modified hydroponic system and on the blotting paper, the corresponding values (number and total length) were significantly (*P* < 0.05) higher in the former. This suggested better growth and development of root system of rice under P+ and P- conditions in the modified hydroponic system compared with the blotting paper. A lower total root length of P+ (3.94 cm ± 0.52 cm [SE]) and P- (3.84 cm ± 0.41 cm [SE]) seedlings during growth on the blotting paper compared with modified hydroponic system further provided evidence toward the efficacy of the latter. Therefore, the modified hydroponic system was employed for subsequent analysis of the effects of Pi deprivation for 4 and 7 days on different root traits.

**FIGURE 6:**
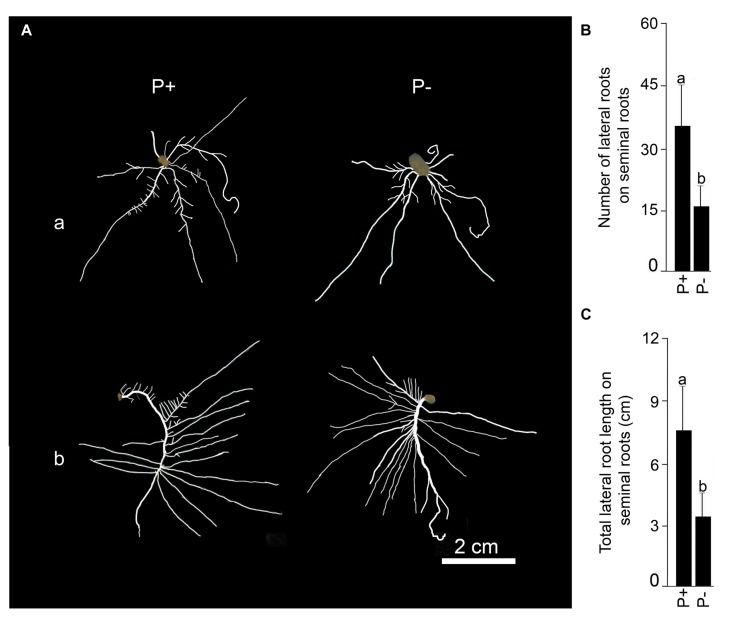
**Effects of Pi deficiency for 4 days on RSA.** MI48 seedlings (4-days-old) were grown under P+ and P- conditions for 4 days. **(A)** Harvested roots were spread to reveal the details of RSA showing (a) seminal and (b) primary roots with well developed lateral roots of both P+ and P- seedlings. Data are presented for **(B)** number and **(C)** total length of lateral roots on seminal roots. Values **(B,C)** are mean ± SE and *n* = 12 replicates. Different letters on the histogram indicate that the means differ significantly (*P* < 0.05).

Seedlings were grown under P+ and P- conditions for 4 days and their RSA are presented in **Figure 6A**. Although, there was no significant (*P* < 0.05) difference in primary root length of P+ (5.22 cm ± 0.84 cm [SE]) and P- (4.57 cm ± 1.39 cm [SE]) seedlings, it was significantly (*P* < 0.05) higher compared with corresponding 2 days seedlings. This revealed a progressive increment in primary root length of both P+ and P- seedlings over a period of time. There was a significant (*P* < 0.05) increase in the percentage of P+ and P- primary roots with well developed lateral roots from ∼25 (2 days) to ∼75 (4 days) suggesting a temporal delay in the development of lateral roots irrespective of Pi regime. However, there were substantial variations in both the number (P+, 28.92 ± 11.35 [SE]; P-, 19.58 ± 6.17 [SE]) and length (P+, 23.23 cm ± 8.91 cm [SE]; P-, 23.06 cm ± 7.47 cm [SE]) of 1st-order lateral roots of P+ and P- seedlings. A similar trend was also observed for the number (P+, 8.42 ± 4.16 [SE]; P-, 3.67 ± 1.89 [SE]) and length (P+, 1.37 cm ± 0.65 cm [SE]; P-, 0.85 cm ± 0.44 cm [SE]) of 2nd-order lateral roots of these seedlings. Therefore, it was not surprising to see a lack of significant (*P* < 0.05) differences in any of these lateral root traits under different Pi regime. The effect of Pi deprivation was also not evident on the number (P+, 8 ± 0.57 [SE]; P-, 8 ± 0.61 [SE]) and total length (P+, 20.29 cm ± 2.99 cm [SE]; P-, 18.33 cm ± 1.88 cm [SE]) of seminal roots. Although, both primary and seminal roots are embryonic in origin, the latter contributed significantly toward the total root length under both P+ and P- conditions. There is a positive correlation between seminal root length and the ability of rice genotypes to produce deep roots and high yield (Rahman and Musa, 2009). Lateral root development on the seminal roots became apparent only 4 days after treatment and both the number and total length were significantly (*P* < 0.05) higher in P+ seedlings compared with P- seedlings (**Figures 6B,C**). Adventitious roots were not detected in these seedlings. Despite some effects of Pi deprivation on the developmental responses of lateral roots on seminal roots, differences in the total root length of P+ (58.01 cm ± 6.55 cm [SE]) and P- (48.81 cm ± 6.21 cm[SE]) seedlings were insignificant (*P* < 0.05).

**FIGURE 7:**
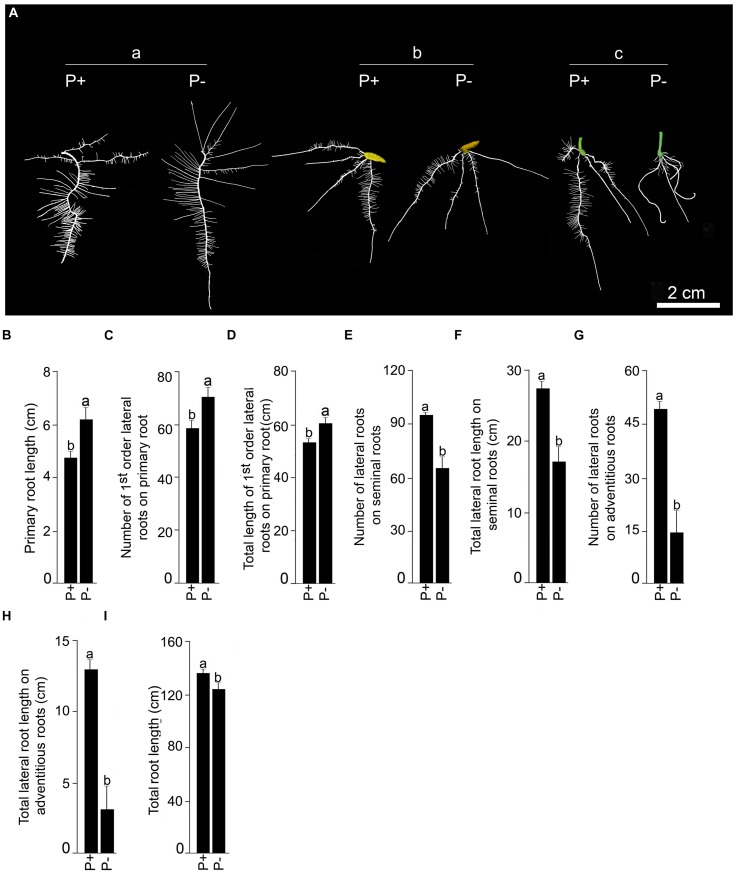
**Effects of Pi deficiency for 7 days on MI48 RSA.** MI48 seedlings (4-days-old) were grown under P+ and P- conditions for 7 days. **(A)** Harvested roots were spread to reveal the details of RSA of P+ and P- seedlings showing (a) primary, (b) seminal and (c) adventitious roots along with their lateral roots. Data are presented for primary root length **(B),** number **(C)**, and total length **(D)** of 1st order lateral roots on primary root, number **(E),** and total length **(F)** of lateral roots on seminal roots, number **(G)** and total length **(H)** of lateral roots on adventitious roots and total root length **(I).** Values **(B–I)** are mean ± SE and *n* = 12 replicates. Different letters on the histogram indicate that the means differ significantly (*P* < 0.05).

**FIGURE 8:**
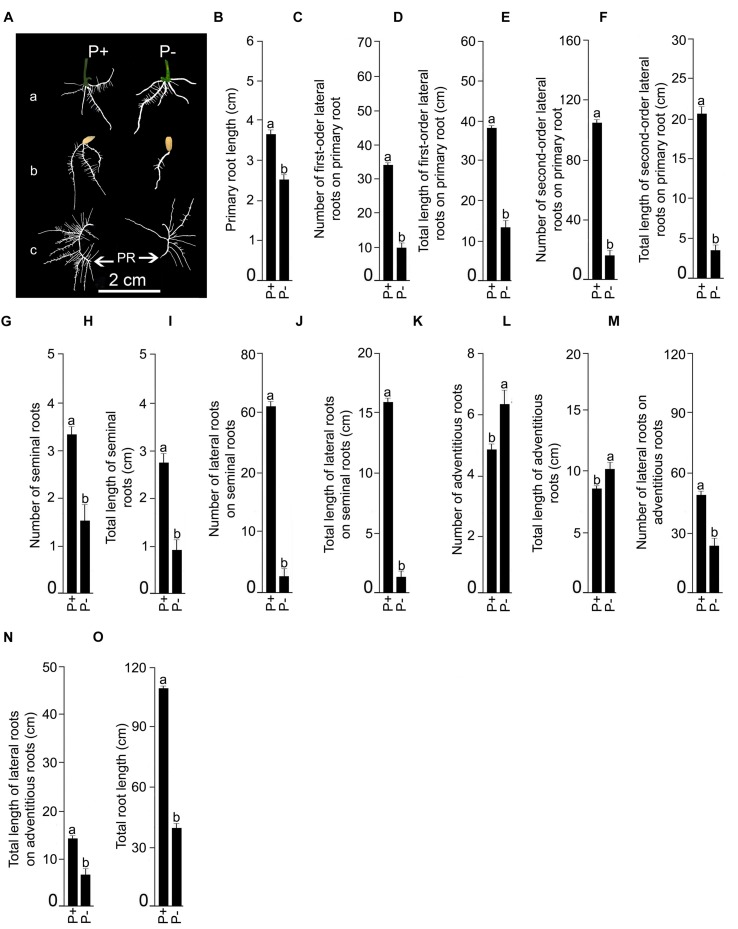
**Effects of Pi deficiency for 7 days on N22 RSA.** N22 seedlings (4-days-old) were grown under P+ and P- conditions for 7 days. **(A)** Harvested roots were spread to reveal the details of RSA of P+ and P- seedlings showing (a) adventitious roots, (b) seminal and (c) primary roots along with their lateral roots. Data are presented for primary root length **(B),** number **(C)**, and total length **(D)** of 1st order lateral roots on primary root, number **(E)** and total length **(F)** of 2nd order lateral roots on primary root, number **(G)** and total length **(H)** of seminal roots, number **(I)** and total length **(J)** of lateral roots on seminal roots, number **(K)** and total length **(L)** of adventitious roots, number **(M)** and total length **(N)** of lateral roots on adventitious roots, and total root length **(O).** Values **(B–O)** are mean ± SE and *n* = 12 replicates. Different letters on the histogram indicate that the means differ significantly (*P* < 0.05).

Finally, the effects of Pi deficiency for 7 days on different RSA traits were determined. Details of P+ and P- RSA are presented in **Figure 7A**. Pi deficiency triggered significant (*P* < 0.05) increase (∼20%) in primary root length compared with P+ seedling (**Figure 7B**). The result was consistent with earlier studies reporting Pi deficiency-mediated accentuated growth response of primary root in *O. sativa* ssp. *indica* genotype Kasalath (Shimizu et al., 2004) and *O. sativa* ssp. *japonica* genotypes Zhonghua10 (Dai et al., 2012, 2016) and Nipponbare (Zhou et al., 2008; Torabi et al., 2009; Zheng et al., 2009; Hu et al., 2011). Interestingly, a similar trend was also observed in NIL6-4 derived from Pi deficiency-intolerant Nipponbare × Pi deficiency-tolerant Kasalath (Torabi et al., 2009). Primary root of P+ and P- seedlings exhibited exuberant growth of lateral roots. There were significant (*P* < 0.05) increases in both the number and total length of 1st-order lateral roots of P- seedlings compared with P+ seedlings (**Figures 7C,D**). The result was consistent with earlier studies on *japonica* genotypes Dongjin and Nipponbare exhibiting augmented number and/or length of lateral roots on primary root of Pi-deprived seedling (Wang S. et al., 2014; Yang et al., 2014). Some of the older 1st-order lateral roots of P+ and P- seedlings developed 2nd-order lateral roots but differences in their number (P+, 52.5 ± 5.99 [SE]; P-, 44.58 ± 4.18 [SE]) and total length (P+, 11.95 cm ± 1.48 cm [SE]; P-, 11.43 cm ± 3.57 [SE]) were insignificant (*P* < 0.05). Differences in the number (P+, 3.75 ± 0.22 [SE]; P-, 4.17 ± 0.52 [SE]) and total length (P+, 13.98 cm ± 0.48 cm [SE]; P-, 16.39 cm ± 1.92 [SE]) of seminal roots were insignificant (*P* < 0.05) under P+ and P-conditions. However, attenuating effects of Pi deprivation were evident on both the number and total length of lateral roots on seminal roots (**Figures 7E,F**). An earlier study has also shown the attenuating effects of Pi deficiency on seminal root length of *O. rufipogon* (wild rice species) and Curinga (*tropical japonica*; Ogawa et al., 2014). There was also development of adventitious roots from hypocotyls of P+ and P- seedlings. Their number (P+, 4.25 ± 0.22 [SE]; P-, 4.08 ± 0.49 [SE]) and length (P+, 11.48 cm ± 1.31 cm [SE]; P-, 8.73 cm ± 0.87 cm [SE]) were not significantly (*P* < 0.05) influenced by Pi status of the nutrient medium. Wang S. et al. (2014) also did not observe any significant effect of Pi deficiency on the number of adventitious root of genotypes Dongjin and Nipponbare. On the contrary, in other studies Pi deficiency was found to exert either inhibitory (genotype Zhonghua10, Dai et al., 2016) or stimulatory (genotype Zhonghua10, Dai et al., 2012; genotype Dongjin, Wang et al., 2015) effects on the developmental responses of adventitious root. This clearly suggested the influence of the genotype on Pi deficiency-mediated effects on the number and/or length of adventitious root. Inhibitory effects of Pi deprivation were evident on both the number and total length of lateral roots on adventitious roots compared with P+ seedlings (**Figures 7G,H**). Overall, total root length of P- seedling was significantly (*P* < 0.05) lower compared with P+ seedling (**Figure 7I**). Further, effects of Pi deficiency for 7 days on different root traits were evaluated in Nagina22 (N22) (**Figure 8**). Details of P+ and P- RSA are presented in **Figure 8A** Pi deficiency exerted attenuating effects on the primary root length (**Figure 8B**), which was consistent with an earlier study (Panigrahy et al., 2014). Interestingly though, the response was contrary to the stimulatory effect on this root trait in MI48. Shimizu et al. (2004) also reported stimulation and no effect on the primary root growth during Pi deprivation in Kasalath (indica) and Gimbozu (japonica), respectively. Pi deficiency also exerted inhibitory effects on both the number (**Figure 8C**) and total length (**Figure 8D**) of 1st-order lateral roots on primary root of N22, which was contrary to MI48. A similar inhibitory influence of Pi deficiency was also evident on the number (**Figure 8E**) and total length (**Figure 8F**) of 2nd-order lateral roots on primary root, and number (**Figure 8G**) and total length (**Figure 8H**) of seminal roots of N22. Comparatively, none of these traits were significantly (*P* < 0.05) affected by Pi deficiency in MI48. Although, Pi deficiency did not have significant influence on both the number and total length of adventitious roots in MI48, values for both these traits were significantly (*P* < 0.05) higher in P- seedlings compared with P+ seedlings of N22 (**Figures 8K,L**). Comparative analysis of the effects of Pi deficiency on different root traits of MI48 and N22 clearly revealed the genotypic differences. In addition, there were attenuating effects of Pi deprivation on the number (**Figures 8I,M**) and length (**Figures 8J,N**) of lateral roots on seminal and adventitious roots and total root length (**Figure 8O**) of N22. A similar effect of Pi deficiency was also observed for these root traits in MI48. The study thus indicated the efficacy of the modified hydroponic system in delineating both differential and comparable effects of Pi deficiency on different root traits in these genotypes.

### Pi Deficiency-Mediated Molecular Responses

**FIGURE 9:**
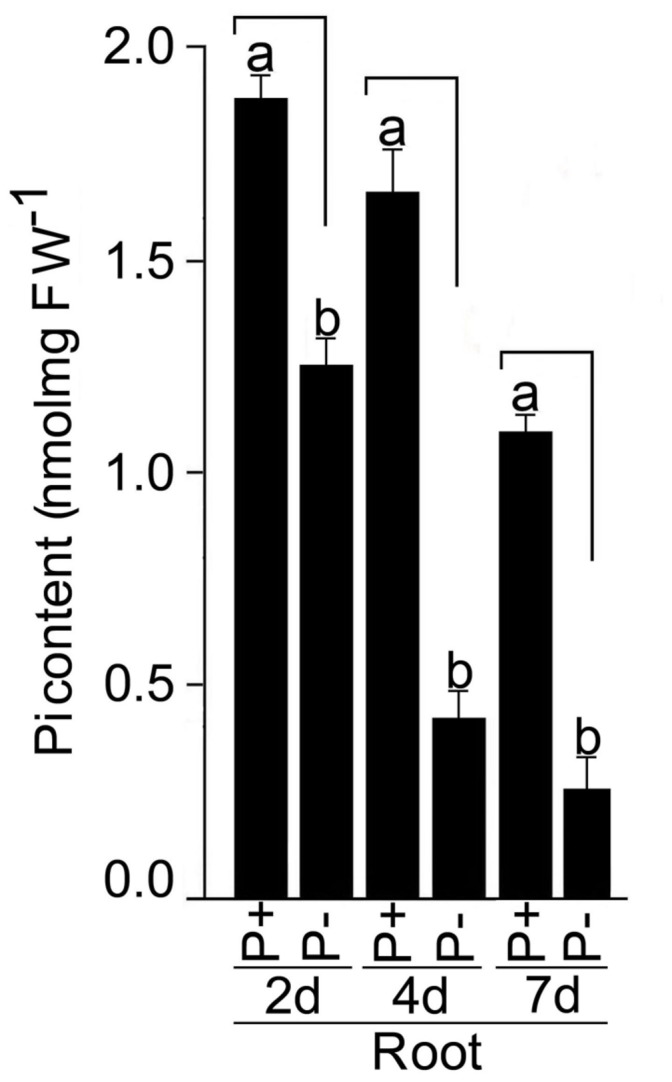
**Effects of Pi deficiency on soluble Pi content.** MI48 seedlings (4-days-old) were grown under P+ and P- conditions for 2, 4, and 7 days. Roots were harvested for determining soluble Pi content. Values are mean ± SE and *n* = 12 replicates. Different letters on the histogram indicate that the means differ significantly (*P* < 0.05).

**FIGURE 10:**
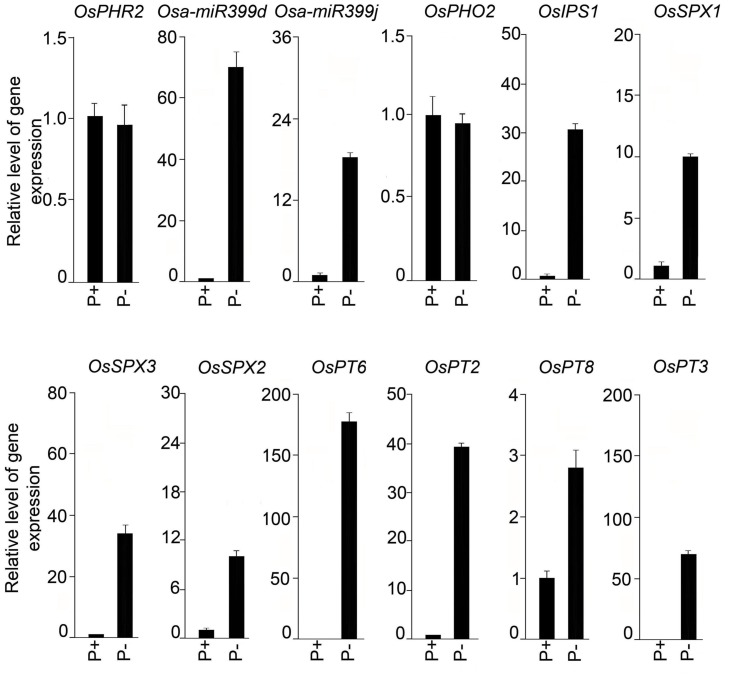
**Effects of Pi deficiency-mediated responses of genes involved in Pi homeostasis.** Real-time PCR analysis of relative expression levels in roots of MI48 seedlings grown under P+ and P- conditions for 7 days. *OsRubQ1* was used as an internal control. Data presented are means of six technical replicates ± SE.

Roots of seedlings grown under P+ and P- conditions for 2, 4, and 7 days were analyzed for soluble Pi content (**Figure 9**). Compared with P+ roots, Pi content in P- roots was significantly (*P* < 0.05) reduced by 33, 73, and 77% after Pi deprivation for 2, 4, and 7 days, respectively. Several genes have been identified in rice that play pivotal roles in the maintenance of Pi homeostasis in rice (Wu et al., 2013). The effects of Pi deficiency on the relative expression levels of these genes in the roots of seedlings grown under P+ and P- conditions for 7 days were assayed by real-time PCR (**Figure 10**). There was no induction of transcription factor *OsPHR2* in response to Pi deficiency and was consistent with an earlier study (Zhou et al., 2008). Whereas, there were 69- and 18-fold induction in the relative expression levels of OsmiR399d and OsmiR399j, respectively. An earlier study had also reported Pi deficiency-mediated induction of OsmiR399s; a pivotal component of Pi sensing and signaling pathway downstream of *OsPHR2* (Zhou et al., 2008). miRNA399 targets E2 ubiquitin-conjugase *OsPHO2*, which is expressed constitutively irrespective of the Pi regime (Hu et al., 2011). Consistent with this report, the relative expression levels of *OsPHO2* were comparable in P+ and P- roots. Further, Pi deficiency triggered 31-fold induction in the relative expression level of *OsIPSI.* Hou et al. (2005) also reported rapid induction of *OsIPSI* in Pi-deprived roots of rice and has been implicated in potentially mimicking OsmiR399 target thereby attenuating its suppressive effect (Wu et al., 2013). Proteins harboring the SPX domain (*OsSPX1–6*) play key roles in the maintenance of Pi homeostasis (Secco et al., 2012). In P- roots, there were significant increases in the relative expression levels of *OsSPX1* (∼10-fold), *OsSPX2* (∼10-fold), and *OsSPX3* (∼34-fold) compared with P+ roots suggesting their roles in the maintenance of Pi homeostasis and were coherent with an earlier study demonstrating their significant induction during Pi deficiency (Wang et al., 2009). SPX1 and SPX2 act as Pi-dependent inhibitors of OsPHR2 activity (Wang Z. et al., 2014). Pi deficiency also triggered significant increases in the relative expression levels of Pi transporters *OsPT2* (∼40-fold), *OsPT3* (∼70-fold), *OsPT6* (∼170-fold), and *OsPT8* (∼3-fold) in P- roots compared with P+ roots. *OsPT2*, a low-affinity Pi transporter, plays a role in mobilizing stored Pi in plants and high-affinity Pi transporter *OsPT6* has been implicated in uptake and translocation of Pi throughout the plant (Ai et al., 2009). *OsPT8*, another high-affinity Pi transporter, is essential for the maintenance of Pi homeostasis and proper growth and development of plant (Jia et al., 2011). *OsPT3* has not yet been functionally characterized.

## Conclusion

The modified hydroponic system was amenable for detailed analysis of the temporal effects of Pi deprivation on the developmental responses of primary, seminal and adventitious roots and also of the lateral roots on each of them of rice genotypes MI48 and N22. The data generated on Pi deficiency-mediated effects on different root traits could be employed for mathematical simulation and modeling. The modified hydroponic system also facilitated generation of tissues for physiological and molecular analyses. It is equally conducive for studying the effects of other nutrient deficiencies or cross talk between different nutrients on morphophysiological and molecular responses of rice genotypes. This modified hydroponic system would also facilitate in rapid identification of Pi deficiency-responsive root traits in a large number of genotypes for genome-wide association study (GWAS).

## Author Contributions

AJ conceived the project and designed the experiments. MN, RS, and VR contributed toward execution of all the experiments. AJ wrote the manuscript. All authors read and approved the final manuscript.

## Conflict of Interest Statement

The authors declare that the research was conducted in the absence of any commercial or financial relationships that could be construed as a potential conflict of interest.

## Footnotes

1 http://rsb.info.nih.gov/ij

2 https://www.idtdna.com
