# Supplementary material for: Deciphering Phosphate Deficiency-Mediated Temporal Effects on Different Root Traits in Rice Grown in a Modified Hydroponic System
Source: Front Plant Sci. 2016 May 4;7:550. doi: 10.3389/fpls.2016.00550 (PMC4855036; doi:10.3389/fpls.2016.00550)
Supplement: Supplementary file 1 [file Table_1.DOC]

Supplementary Table 1. Primer sequences for Real-time PCR analysis

| Gene | Gene Identifier | Primer sequence (5'→3') | Product (bp) |
| --- | --- | --- | --- |
| *OsPT2* | AF536962 | F: GACGAGACCGCCCAAGAAG | 74 |
|  |  | R: TTTTCAGTCACTCACGTCGAGAC |
| *OsPT3* | AF536963 | F: TGCGACTGCTGTATTCAGTACGT | 126 |
|  |  | R: ACAAATGCCATCAAATATGAACAGA |
| *OsPT6* | AF536966 | F: TATAACTGATCGATCGAGACCAGAG | 76 |
|  |  | R: TGGATAGCCAGGCCAGTTATATATC |
| *OsPT8* | AF536968 | F: AGAAGGCAAAAGAAATGTGTGTTAAAT | 114 |
|  |  | R: AAAATGTATTCGTGCCAAATTGCT |
| *OsSPX1* | [Os06g0603600](http://rapdb.dna.affrc.go.jp/viewer/gbrowse_details/irgsp1?name=Os06g0603600) | F: ACATTTGCTGGAGATAGTACCG | 145 |
|  |  | R: TCTGATGGTTATGATGGGTTTCT |
| *OsSPX2* | [Os02g0202200](http://rapdb.dna.affrc.go.jp/viewer/gbrowse_details/irgsp1?name=Os02g0202200) | F: CTCAATGTGCAGCCTGACTA | 91 |
|  |  | R: CAGCCATACAAACCATCTAACAAA |
| *OsSPX3* | [Os10g0392600](http://rapdb.dna.affrc.go.jp/viewer/gbrowse_details/irgsp1?name=Os10g0392600) | F: TGCCGGTACTAGATTGTATGATT | 110 |
|  |  | R: GTATGTTCTCTACCACGGCATA |
| *OsPHR2* | [Os07g0438800](http://rapdb.dna.affrc.go.jp/viewer/gbrowse_details/irgsp1?name=Os07g0438800) | F: GACCAGAATTGTCTGAAGGTTCTT | 107 |
|  |  | R: ACGCAATGCCTCAGTGAGAT |
| *OsPHO2* | Os05g0557700 | F: CGAGAATTTTGTCAAGGAGCA | 99 |
|  |  | R: TCACGAGCATGTCCAACAA |
| *OsIPS1* | AY5687591 | F: CTAAGGTAGGGCAACTTGTATC | 182 |
|  |  | R: TTATTAGAGCAAGGACCGAAAC |
| *OsmiR399d* | MI0001056 | F:GGTGGCCTTTGATAGACCATCA | 116 |
|  |  | R:GCAGGCCGTTTTGGTGAAT |
| *OsmiR399j* | MI0001062 | F:GGAGCATGTGAAGTCTTTTGTAGC | 61 |
|  |  | R:GGCAACTCTCCTTTGGCAGA |
| *OsRubQ1* | Os06g0681400 | F: GGGTTCACAAGTCTGCCTATTTG | 75 |
|  |  | R: ACGGGACACGACCAAGGA |
